# Generation of a malaria negative Ugandan birth weight standard for the diagnosis of small for gestational age

**DOI:** 10.1371/journal.pone.0240157

**Published:** 2020-10-02

**Authors:** Arthurine K. Zakama, Terik Weekes, Richard Kajubi, Abel Kakuru, John Ategeka, Moses Kamya, Mary K. Muhindo, Diane Havlir, Prasanna Jagannathan, Grant Dorsey, Stephanie L. Gaw

**Affiliations:** 1 Division of Maternal-Fetal Medicine, Department of Obstetrics, Gynecology, and Reproductive Sciences, University of California, San Francisco, California, United States of America; 2 Infectious Diseases Research Collaboration, Kampala, Uganda; 3 Department of Medicine, Makerere University, Kampala, Uganda; 4 Department of Medicine, University of California, San Francisco, California, United States of America; 5 Department of Medicine, Stanford University, Stanford, California, United States of America; University of Cambridge, UNITED KINGDOM

## Abstract

**Objective:**

Placental malaria is a known risk factor for small for gestational age (SGA) neonates. However, currently utilized international and African birthweight standards have not controlled for placental malaria and/or lack obstetrical ultrasound dating. We developed a neonatal birthweight standard based on obstetrically dated pregnancies that excluded individuals with clinical malaria, asymptomatic parasitemia, and placental malaria infection. We hypothesized that current curves underestimate true ideal birthweight and the prevalence of SGA.

**Study design:**

Participants were pooled from two double-blind randomized control trials of intermittent preventive therapy during pregnancy in Uganda. HIV-negative women without comorbidities were enrolled from 12–20 weeks gestation. Gestational age was confirmed by ultrasound dating. Women were followed through pregnancy and delivery for clinical malaria, asymptomatic parasitemia, and placental malaria. Women without malaria, asymptomatic parasitemia, or placental malaria formed the malaria negative cohort and generated the Ugandan birthweight standard. The Ugandan standard was then used to estimate the prevalence of SGA neonates in the malaria positive cohort. These findings were compared to international (Williams, World Health Organization (WHO), and INTERGROWTH-21st) and regional standards (Tanzanian and Malawi).

**Results:**

926 women had complete delivery data; 393 (42.4%) met criteria for the malaria negative cohort and 533 (57.6%) were malaria positive. The Ugandan standard diagnosed SGA in 17.1% of malaria positive neonates; similar to the INTERGROWTH-21^st^ and Schmiegelow curves. The WHO curve diagnosed SGA in significantly more neonates (32.1%, p = <0.001), and the Malawi curve diagnosed SGA in significantly fewer neonates (8.3%, p <0.001).

**Conclusion:**

Exclusion of women with subclinical placental malaria in malaria-endemic areas created birth weight norms at higher values and increased the detection of SGA. Birth weight standards that fail to account for endemic illness may underestimate the true growth potential of healthy neonates.

## Introduction

Small for gestational age (SGA) is defined as neonates weighing less than the 10^th^ percentile of birth weight for a specific completed gestational age of a given reference population [1]. SGA is associated with a high risk of neonatal morbidity and mortality [1–3]; thus, appropriately characterizing at-risk fetuses and neonates is paramount. However, research has found that the prevalence of SGA in a given population can vary significantly based on the reference cohort utilized [2]. Studies have found that international standards such as the WHO, may underestimate growth in well-resourced countries while overestimating growth for low-resourced nations [4]. Even within a given country, there can be significant differences between racial and ethnic groups in relation to the prevalence of SGA [5]. This point of ethnic variation in growth is contested by the INTERGROWTH-21^st^ project that created an international fetal and neonatal growth standard based on the assumption of no difference in global fetal growth development [5,6].

The African continent holds the most ethnic and genetic diversity in the world [7]. Thus, when evaluating the prevalence of SGA in African countries, the debate regarding the presence of ethnic variety in fetal growth is at the forefront. However, available international birth weight standards either sample a few countries on the continent or adapt formulas and curves based on an all-Caucasian population. Additionally, many prominent standards [8,9] have not used obstetrical ultrasound to confirm gestational age—lacking optimal pregnancy dating records [10].

Many low-resource countries in Africa have endemic infectious diseases, such as malaria, that directly increase the risk of SGA [11,12]. Greater than 25 million pregnant women and their newborns in endemic areas are affected by malaria yearly. Placental malaria is the primary route that malarial infection during pregnancy causes adverse perinatal outcomes [11,13,14]. This occurs through *Plasmodium falciparum* parasites sequestering in the placenta; leading to a disruption in the nutritional exchange between mother and fetus through the generation of local inflammatory responses and decreased blood flow [11,13,15–17]. This chain of events and the associated negative perinatal outcomes can occur even in mothers that are asymptomatic for malaria [18]. Thus, it is possible to have neonates that are SGA as a result of placental malaria without clinical maternal infection. The gold standard for the diagnosis of placental malaria is by histopathologic scoring [13], which has not been regularly performed in any other published growth curve [3,6,8,9,19,20]. Placental histopathology may be important to account for in the generation of normal curves, as up to 10% of pregnancies in malaria-endemic regions have evidence of placental involvement, but no clinical malaria or asymptomatic parasitemia documented in pregnancy [12].

Considering this frequently occult infection and the debate on ethnicity and fetal growth, we sought to evaluate birth weight standards that could define the prevalence of SGA for Uganda. We found that available international and African birth weight standards did not regularly account for placental malaria. We sought to develop a neonatal birth weight standard based on obstetrically dated pregnancies that excluded clinical malaria, asymptomatic parasitemia, and placental malaria infection. We hypothesized that currently available growth curves underestimate true birth weight and the prevalence of SGA. To do this, we generated a growth curve based on a malaria negative Ugandan cohort and compared that to international (Williams, World Health Organization (WHO), and INTERGROWTH-21st) and regional standards (Tanzanian and Malawi).

## Materials and methods

### Study procedures

This study utilizes longitudinally collected data from two double-blind, randomized controlled trials of intermittent preventive therapy during pregnancy in Uganda. The Dihydroartemisinin–Piperaquine for the Prevention of Malaria in Pregnancy study (NCT02163447) enrolled pregnant women from June to October 2014 and randomized them to preventive therapy with sulfadoxine-pyrimethamine given every 8 weeks, dihydroartemisinin-piperaquine given every 8 weeks, or dihydroartemisinin-piperaquine given every 4 weeks during pregnancy [21]. The primary endpoint of this study was the prevalence of histopathologically confirmed placenta malaria. The monthly sulfadoxine–pyrimethamine versus dihydroartemisinin–piperaquine for intermittent preventive treatment of malaria in pregnancy trial (NCT02793622) enrolled pregnant women between September 2016 to May 2017. They were randomly assigned to receive monthly sulfadoxine–pyrimethamine or monthly dihydroartemisinin–piperaquine for intermittent preventive treatment of malaria in pregnancy [22]. The primary endpoint of this study was to evaluate the risk of adverse birth outcomes in each cohort. All participants in both studies were HIV-negative women at least 16 years of age with a viable pregnancy between 12 and 20 weeks gestation.

Gestational age was determined by the first day of the last menstrual period (LMP) and an ultrasound performed on the day of enrollment. Per study protocol [21,22], if the ultrasound was consistent with a gestational age of 6 to 12 weeks and the ultrasound dating was within 7 days of the LMP, then the LMP was used to determine gestational age; if the ultrasound differed from the LMP by more than 7 days, then the ultrasound was used to determine gestational age. If the ultrasound was consistent with a gestational age of 13–24 weeks, and the ultrasound gestational age was within 14 days of the LMP, then the LMP was used to determine gestational age; if the ultrasound gestational age differed from the LMP by more than 14 days, then the ultrasound was used to determine gestational age.

In both studies, routine visits were conducted every 4 weeks. Routine laboratory testing was performed every 8 weeks for complete blood count and alanine aminotransferase. Women were encouraged to come to the clinic any time they felt ill. Those who presented with a documented fever (tympanic temperature ≥38·0°C) or history of fever in the previous 24 hours had blood collected for a thick blood smear for detection of malaria parasites. Blood smears were stained with 2% Giemsa and read by experienced laboratory technologists. A blood smear was considered to be negative when the examination of 100 high-power fields did not reveal asexual parasites. If the smear was positive, the patient was diagnosed with malaria and treated with artemether–lumefantrine. Collection of dried blood spots for molecular detection of asymptomatic parasitemia with quantitative PCR (qPCR) and loop-mediated isothermal amplification (LAMP) of *P*. *falciparum* DNA occurred every 4 weeks per study protocol. Details of how these molecular tests were operated and copy number of the parasite genome have been described for this dataset in previous studies and their protocols [21–23]. The results of these tests were not available until after completion of the study. Thus, asymptomatic parasitemia was not treated. Women were encouraged to deliver their babies at the hospital adjacent to the study clinic. Women delivering at home were visited by study staff at the time of delivery or as soon as possible afterwards. At delivery, a standardized assessment was completed including evaluation for congenital anomalies, birth weight, and collection of biological specimens including placental tissue, placental blood, and maternal blood. Following delivery, women were followed for 6 weeks postpartum.

For assessment of placental malaria, two 1 cm-wide full thickness biopsies, obtained about 5 cm from the cord, were obtained within 1 hour of delivery and placed in 10% neutral buffered formalin. Biopsy specimens were embedded in paraffin wax, sectioned into 3 μm slices using a rotary microtome, fixed to glass slides, and dehydrated in sequential ethanol baths. Separate slides were stained in 0.1% hematoxylin/1% eosin (H&E) for 5 and 1 min, respectively, or in 2% Giemsa for 30 min. Placentas were graded into 5 categories using a standardized Rogerson criteria [24]. The presence of intervillous parasite-infected erythrocytes and of pigment in monocyte/macrophages or fibrin were noted. Quantitative assessments of placental malaria involved counting of 1000 intervillous blood cells under high power and determination of percentages of intervillous infected erythrocytes and monocyte/macrophages containing malarial pigment.

### Cohort definition

Data was analyzed to create the following two cohorts from live, singleton births: malaria negative and any-malaria. The malaria negative cohort was defined as the absence of clinical malaria, asymptomatic parasitemia, and placental malaria. The any-malaria cohort included women with any of the following: clinical malaria, placental malaria, or asymptomatic parasitemia. Placental malaria was diagnosed by positive histopathology by Rogerson criteria [13], with or without symptomatic malarial infection. Asymptomatic parasitemia was defined as no clinical maternal malaria episodes but parasite DNA was detected in the blood by positive peripheral maternal blood qPCR or LAMP, with or without placental malaria.

The malaria negative cohort was used to create a sex-stratified birth weight standard. This birth weight standard was then used to determine the prevalence of SGA neonates, defined as <10% percentile, in the any-malaria cohort. The performance of this birth weight standard was then compared to six commonly used growth curves including: Schmiegelow [20] (based on a Tanzanian population), the WHO global reference (based on Mikolajczyk et. al algorithm) [19],Williams’ US-based curve [9], Verhoeff’s Malawi standard [8], INTERGROWTH-21^st^ (based on a multinational standard) [6], and WHO sex-stratified (based on an international standard detailed in Kiserud’s study [3]). We input our Ugandan specific data into the WHO global reference algorithm as detailed in Mikolajczyk’s paper [19]; the output was used in the above comparison and was labeled as WHO Uganda. The sex-stratified WHO curves based on Kiserud’s study were labeled WHO International.

The six comparison curves were chosen to compare our curve to regional standards (Tanzanian and Malawi), internationally inclusive standards (WHO and INTERGROWTH-21st), and internationally utilized curves from high-income countries (Williams). We aim to use these curves to provide commentary on the design of global birth weight standards and the generalizability of these standards.

### Data analysis

Data were triple-checked and validated using Microsoft Excel 2019. The growth standard chart and curve were created using MathWorks MATLAB R2018b (The MathWorks, Inc., Natick, Massachusetts, United States).

The dataset was organized by completed gestational week in ascending order. For each completed gestational week, the birth weights were arranged in arrays of ascending order. A percentile function similar to the estimation-maximization model approach [25] was implemented due to its ability to estimate the distributions directly. Given our sample size, we opted for this model approach to limit the influence of outliers.

The percentile birth weights derived from our model were smoothed using the MATLAB curve fit tool to create the Ugandan standard growth curve, highlighting the 10th, 50th, and 90th percentiles. A 3^rd^ order polynomial regression was applied to create the growth curve using the least actual residual model.

A function was created to generate the proportion of SGA determined using our Ugandan standard for each cohort of interest incorporating sex-stratification. Using this SGA comparison function we were able to compare the proportion of SGA as determined by each of the six growth standards of interest.

Mann-Whitney U test was used to compare medians of non-parametric continuous variables and chi-squared analysis was used to compare proportions of non-parametric categorical variables. Wilcoxon signed rank test was conducted to compare the diagnosis of SGA by each curve and the percent agreement, relative risk and 95% confidence intervals (CI) were calculated. Statistical analysis was done in SPSS (IBM SPSS Version 23 Armonk, NY: IBM Corp). Forest plots and bar graphs were created in GraphPad Prism Version 8 (GraphPad Software, La Jolla California USA).

### Ethical approval

The two randomized control trials were approved by the ethics committees of Makerere University School of Biomedical Sciences (Kampala, Uganda), the Uganda National Council for Science and Technology (Kampala, Uganda), and the University of California San Francisco (San Francisco, CA, USA). All study participants provided written informed consent.

## Results

**Fig 1:**
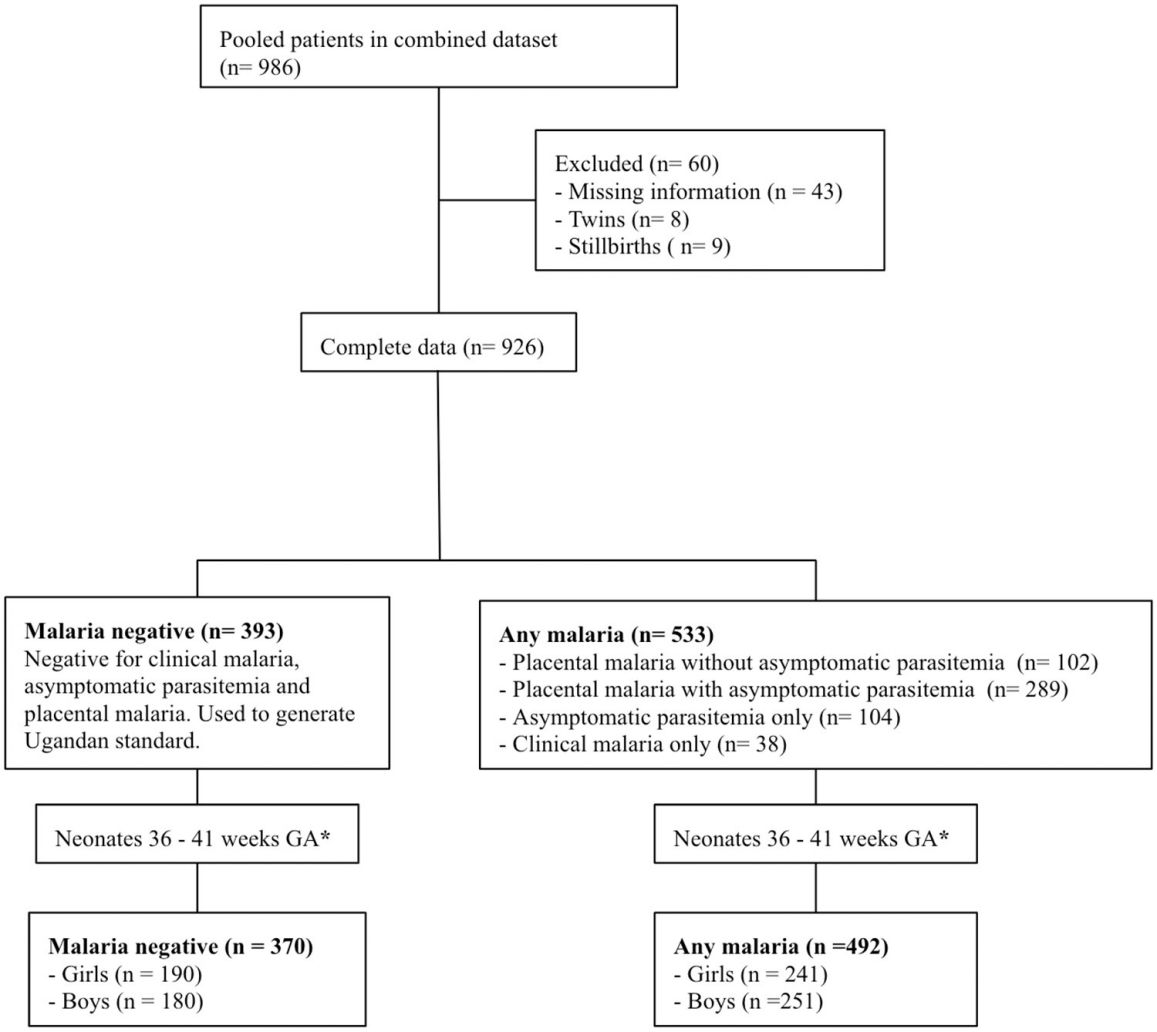
Patient flow chart.

There were a total of 926 women with complete delivery data including gestational age, birth weight, clinical and placental malaria status recorded. From this group, 393 (42.4%) met criteria for the malaria negative group and 533 (57.6%) in the any-malaria group. The malaria negative group was older, had less primigravidas, and had larger birth weight neonates than the any malaria cohort (Fig 1 and Table 1). The sample size per gestational week was limited at the extremes of gestational age; based on this the analysis was limited to 36–41 weeks gestational age to limit the outliers of gestational age with minimal data points (S1 Table).

**Table 1:** Baseline characteristics of cohorts.

Characteristics	Malaria negative (n = 370)	Any malaria (n = 492)	P value*
Maternal age in years, median	25.0	20.8	**<0.001**
Primigravida, n (%)	32 (8.6%)	195 (39.6%)	**<0.001**
GA in weeks at delivery, median	39.9	39.7	0.611
Birth weight in grams, median	3100	3000	**<0.001**
Female newborn, n (%)	190 (51.4%)	241 (50.0%)	0.491
IPTp with DP, n (%)	255(68.9%)	221 (44.9%)	**<0.001**

* Median non-parametric values compared with Mann-Whitney U test. Proportions compared with chi-squared test.

Abbreviations: GA, gestational age; IPTp, intermittent preventative therapy in pregnancy; DP, dihydroartemisinin-piperaquine (DP)

The malaria negative cohort was used to generate the Ugandan standard. The any-malaria cohort was subdivided into placental malaria (including those with and without asymptomatic parasitemia), asymptomatic parasitemia (including those with and without placental malaria), and clinical malaria. Statistical analysis was done on the any-malaria group 36–41 weeks gestation (n = 492).

**Fig 2:**
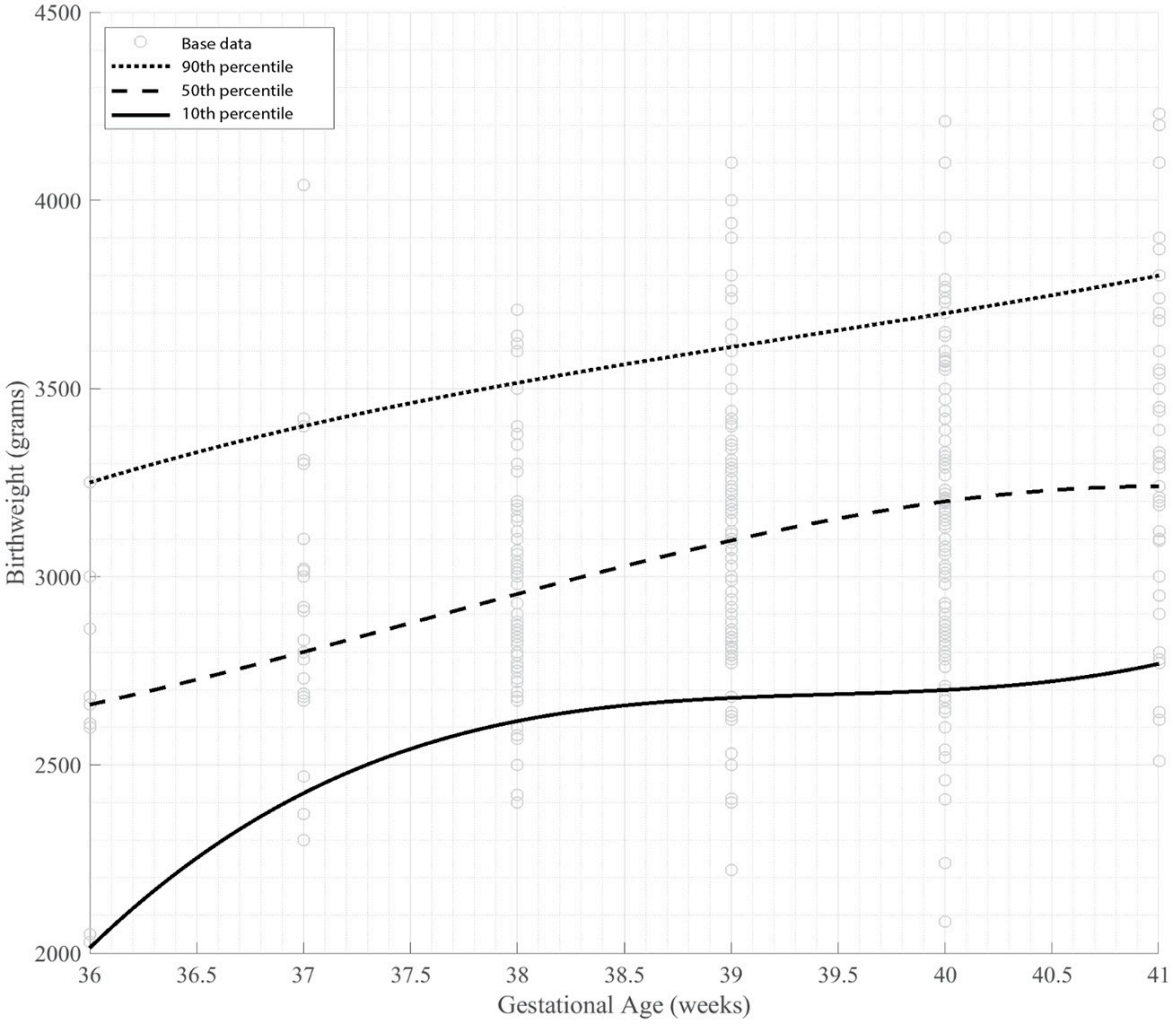
Malaria negative birth weight curve.

The malaria negative cohort was used to generate the Ugandan birth weight standard. This was then stratified by sex to generate the Ugandan standard for girls and boys. The birth weight curve and tables are listed in Fig 2 and Table 2.

**Table 2:** Malaria negative standard GA by birth weight.

	Birth Weight Percentile (grams)								
	Non-Stratified (n = 370)			Girls (n = 190)			Boys (n = 180)		
GA	10th	50th	90th	10th	50th	90th	10th	50th	90th
36	2030	2660	3250	2030	2660	3000	2050	2680	3250
37	2370	2800	3400	2300	2920	3100	2470	2780	3420
38	2670	2900	3400	2580	2850	3400	2730	2980	3300
39	2680	3180	3630	2630	3100	3420	2780	3230	3800
40	2670	3200	3700	2670	3195	3580	2680	3200	3700
41	2780	3240	3800	2770	3200	3800	2780	3330	3900

Gestational age presented as completed weeks. Birth weight presented in grams. GA: Gestational age.

The malaria negative cohort, 36 to 41 weeks (n = 370), was used to create the malaria negative birth weight curve. The 10^th^, 50^th^, and 90^th^ percentiles were smoothed graphically with 3rd order polynomial regression through the least actual residual model in MathWorks MATLAB R2018b (The MathWorks, Inc., Natick, Massachusetts, United States).

Of the studies involved in the comparison, all studies had obstetrical ultrasound dating with the exception of Williams’ United States [9] and Verhoeff’s Malawi [8] curves; both of these curves used LMP as the primary means of dating. Kiserud’s WHO curve [3], Williams [9], and Verhoeff’s Malawi [8] curves did not exclude the diagnosis of malaria from the cohort used to create their curves. The INTERGROWTH-21^st^ [6] curve excluded women with episodes of malaria; however, the exact testing used for this exclusion was not discussed in their report. Of the curves used in the comparison, Schmiegelow [20], WHO Uganda [19], and Williams’ [9] curves did not stratify based on sex, the remaining curves stratified by sex. See Table 3 for detailed population characteristics across studies.

**Table 3:** Comparison of population characteristics across studies.

	Birth Weight Curve						
Characteristics	Ugandan	Schmiegelow [20]	WHO Uganda [19]	Williams [9]	Intergrowth [6]	Verhoeff [8]	WHO International [3]
Population (n)	Uganda (370)	Tanzania (583)	Multinational (237,025)	United States (37,862)	Multinational (4321)	Malawi (1423)	Multinational (1274)
Maternal Age	25.9 +/- 5.7	27.1 +/- 6.2	---	---	28.4 +/- 3.9	---	28*
Primigravida (n (%))	32 (8.6%)	99 (17%)	---	---	2955 (68%)	---	739 (58%)
GA at Delivery (weeks)	39.9	40.1	---	---	---	38.6^#^	39.4
Birth weight (grams)	3100	3170	---	---	---	2818^#^	3300
Accounts for Sex	Yes	No	No	No	Yes	Yes	Yes
Malaria Excluded	Yes	Yes	No	No	Yes	No	No
Type of Malaria Testing^+^			---	---	---		---
Clinical malaria	BS,LAMP	---				---	
Asymptomatic parasitemia	BS,LAMP, qPCR	BS, RDT				BS	
Placental malaria	HP, BS, LAMP	---				BS	
Dating Ultrasound	Yes	Yes	Yes	No	Yes	No	Yes

The population number is listed in the table as the number of participants used to generate the birth weight standard. Maternal age presented as mean +/- standard deviation unless otherwise indicated. Median gestational age presented in weeks and birth weight presented in grams unless otherwise indicated. ---indicates that a metric was not reported. Abbreviations GA: gestational age | BS: blood smear | HP: histopathology | LAMP: loop mediated isothermal amplification | RDT: rapid diagnostic test | qPCR: quantitative polymerase chain reaction

*Median maternal age.

^#^Mean.

^+^Method of diagnosis for each category of malarial infection is listed.

**Fig 3:**
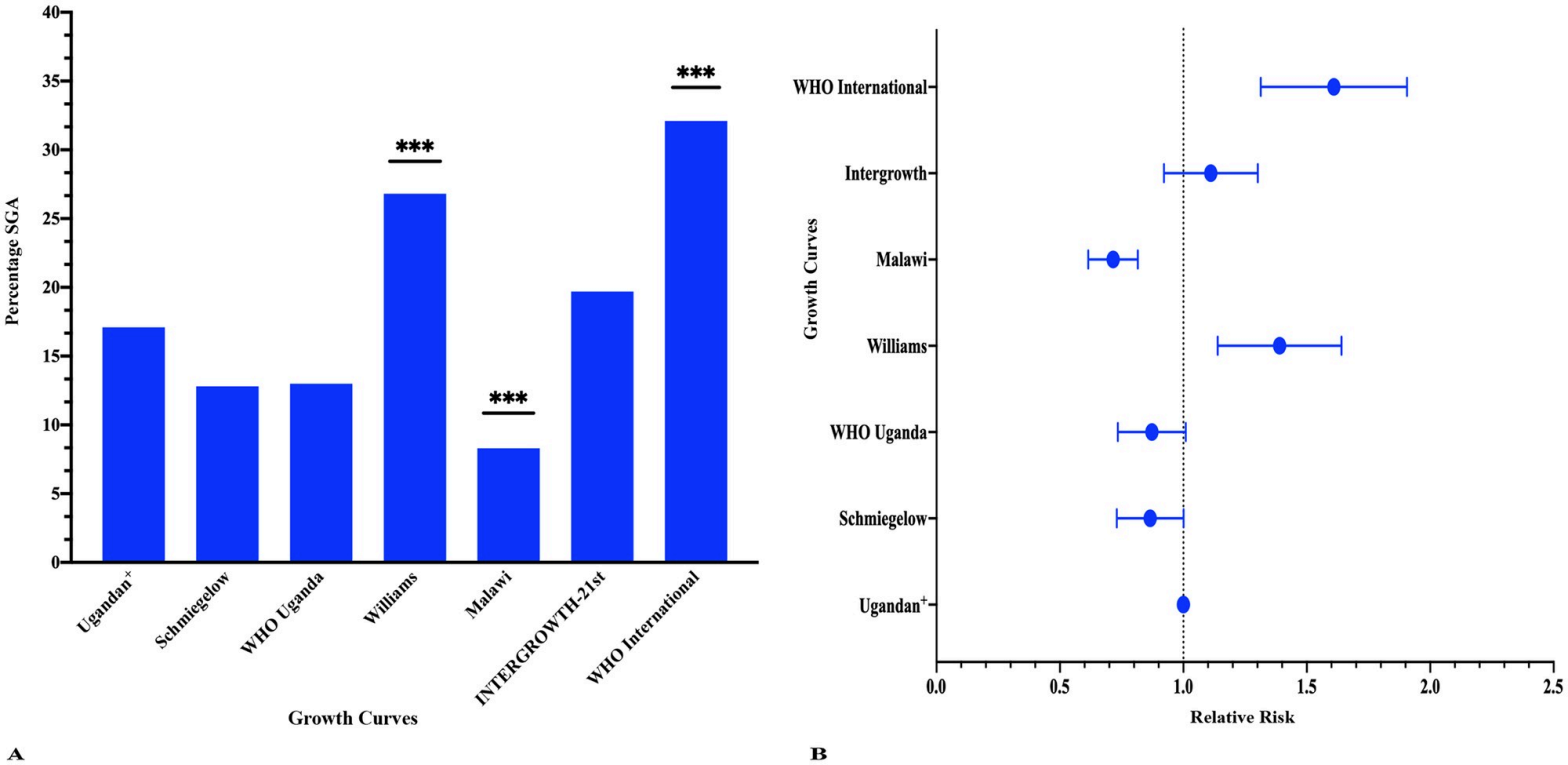
Proportion SGA as determined by growth curves. (A) Percentage SGA by growth curves. (B) Forest plot of relative risk in growth curves. Proportion small for gestational age (SGA) calculated for the any-malaria cohort for each growth curve. Wilcoxon signed rank test and relative risk were calculated to assess statistical difference between the diagnosis of SGA between curves for 36–41 weeks’ gestation. Sex stratification was applied for the Ugandan, Malawi, INTERGROWTH-21^st^ and WHO International curves. ^+^ Malaria negative reference. *** p < 0.001.

The Ugandan curve diagnosed SGA in 17.1% of the any-malaria cohort. This was slightly more than Schmiegelow (12.8%) and WHO Uganda (13.0%), but these findings did not reach statistical significance (p = 0.07 and p = 0.08 respectively; percent agreement for SGA 67.2% and 51.8% respectively). INTERGROWTH-21^st^ diagnosed SGA in 19.7% of the any-malaria cohort; this was not statistically different from the Ugandan curve (p = 0.07; percent agreement of SGA 48.4%). Williams and WHO International curves diagnosed significantly more neonates as SGA compared to the Ugandan curve (26.8%, p <0.001 and 32.1%, p = <0.001 respectively; percent agreement 49.0% and 20.0% respectively). Verhoeff’s Malawi curve diagnosed significantly less neonates as SGA as the Ugandan curve (8.3%, p = <0.001; percent agreement of SGA 35.0%). See Fig 3 and Table 4 for details of the statistical comparisons.

**Table 4:** Percent agreement between growth curves.

Any Malaria (n = 492)		
	SGA (n (%))	Percent Agreement
Ugandan	84 (17.1%)	(reference)
Schmiegelow [20]	63 (12.8%)	67.2%
WHO Uganda [19]	64 (13.0%)	51.8%
Williams [9]	132 (26.8%)	49.0%
Malawi [8]	41 (8.3%)	35.4%
INTERGROWTH-21^st^ [6]	97 (19.7%)	48.4%
WHO International [3]	158 (32.1%)	20.0%

For each curve, the percent agreement for SGA was calculated.

## Discussion

### Principal findings

We created a malaria negative birth weight standard based on longitudinally assessed maternal malaria status and histopathologic placental evaluation for assessing clinical malaria, placental malaria, and asymptomatic parasitemia. We found that our malaria negative Ugandan standard tended to diagnose more SGA neonates than WHO Uganda and Schmiegelow’s Tanzanian curve in women with malaria infection. The INTERGROWTH-21^st^ curve performed similarly to the Ugandan standard in diagnosing SGA while the WHO International curve statistically overestimated SGA in neonates with maternal malaria infection.

### Comparison with existing literature

Here we present the first birth weight standard that definitively excluded malaria infection (clinical malaria, placental malaria, and asymptomatic parasitemia) during pregnancy, a significant cause of low birth weight and fetal growth restriction. As supported by previous research, the malaria negative cohort was older, had more multigravidas, larger neonates, and used dihydroartemisinin-piperaquine for IPTp instead of sulfadoxine-pyrimethamine when compared to the any malaria cohort [11,21]. The creation of a birth weight standard based on a true malaria negative cohort has not previously been done as birth weight standards in malaria-endemic regions have not assessed for malarial infection (particularly by placental histopathology), purposefully included malaria positive patients, or only assessed the serum for parasitemia [3,6,8,19,20]. This is important and novel because placental malaria is the known linkage between maternal malarial infection and adverse obstetrical outcomes such as low birth weight (relative risk (RR), 3.45; 95% CI 1.44–8.23; p = 0.005), preterm birth (RR, 7.52; 95% CI 1.72–32.8; p = 0.007), small for gestational age (RR, 2.30; 95% CI 1.10–4.80; p = 0.03), and higher rates of maternal anemia (adjusted odds ratio, 2.22; 95% CI 1.02–4.84; p = 0.045) [11,12,14].

We found that Williams’s US-based curve overestimated SGA and fetal growth. This finding is congruent with some of the current literature. One study showed that birth weight standards based on high-income countries often overestimate growth in Sub-Saharan Africa leading to the predication of larger neonates than what is accurate [26]. However, this study did not exclude maternal malaria or HIV infection in the cohort used to create their population standard [26]. Their finding of smaller neonates with the inclusion of endemic diseases aligns with the underdiagnosis of SGA we found in the Malawi curve which screened for peripheral and placental malaria, but did not exclude women who were positive for malaria [8]. We created a malaria and HIV negative cohort to assess optimal growth in the Ugandan population. Our findings aligned with INTERGROWTH-21^st^, even with lower percent agreement, which sought to globally evaluate fetal and neonatal growth in low-risk pregnancies. The discrepancies here highlight that international standards can be applicable if they include diverse patient populations, INTERGROWTH-21^st^ represented a multinational population while Williams’ completed curve is on a United States-only population [9]. Additionally, the decision to exclude or include endemic diseases impacts the population standard as it defines what “normal” is. We believe normal growth in Sub-Saharan Africa can be achieved in the absence of the disease processes currently disproportionately affecting the region and should be represented in the medical literature.

Our findings also add to the growing discussion on the types of birth weight standards and references (web-based algorithms) used to define SGA. Gardosi et al, have argued for the creation of a customized birth weight standard, based on individual maternal characteristics instead of population-based standards [27,28]. They created an algorithm where maternal height and weight, ethnicity, parity, fetal weight and sex, and gestational age are analyzed to produce a customized birth weight centile [2,28]. Many studies have investigated their approach to assess its validity. One meta-analysis found that the customized charts estimated higher odds ratios for mortality in SGA neonates in comparison to population-based charts; however, these differences had overlapping confidence intervals [29]. Another comparison between Gardosi’s customized model and INTERGROWTH-21^st^ population-based standard found that INTERGROWTH-21^st^ both overestimated and underestimated SGA in healthy cohorts from 10 countries, while the customized curve was closer to the tenth percentile for most of the cohorts [30]. These findings have raised doubts about the one-size fits all approach to population-based fetal growth standards [30].

However, these studies primarily evaluated the above birth weight standards in high-income countries and excluded African countries. Africa is home to the most ethnic diversity globally [7]; thus, validating birth weight standards on the continent is paramount. Care must be taken when including African cohorts to note the ethnic diversity that exists within countries given the history of continental migration and the creation of national borders primarily through colonialism. Through this lens, we recommend for the exclusion of endemic diseases in order to more accurately assess fetal growth, an approach demonstrated to have a higher sensitivity for identifying SGA [19,20].

### Clinical implications

Our findings highlight the importance of a globally inclusive patient population when creating a birthweight standard. We found that the WHO International, based on 10 countries, overestimated fetal growth in our cohort and had the lowest percent agreement of 20%. The cohort in their study was 57.8% Caucasian [3]. However, the WHO Uganda curve, based on the WHO’s global reference, was similar to our Ugandan standard but had a percent agreement of just 51.8%, which may reflect the additional considerations of sex and malaria in the Ugandan standard. The WHO’s global reference algorithm is a combination of data collected and validated from 24 countries, covering Africa, Latin America and Asia, and the utilization of Hadlock’s fetal growth equation [19]. These findings illustrate that diversity in the creation of global reference algorithms (used to insert local data) and standards is important. Based on this, we find, similar to Gardosi [27], that a growth reference algorithm may be more applicable than a population standard. However, we found that a growth reference can be global, if the populations included are representative of the global community; thus, customized curves to each individual may not be needed. Additionally, customized curves requiring multiple data points on maternal characteristics may not be practical in low- and middle-income countries where that information may not be readily available.

With an applicable global reference, low- and middle-income countries can utilize appropriate data to categorize which neonates are SGA and therefore at high risk of morbidity and mortality. Interventions designed to alleviate these risks will have better population data on the magnitude of the issue, highlighting the larger population of neonates that require assistance. Furthermore, allocation of resources can adequately be restructured when the scope of the epidemiological impact of SGA is better defined.

Our study also addresses the exclusion of placental malaria infection, in malaria-endemic regions, for the purpose of obtaining a healthy growth standard. Schmiegelow’s curve, based on a population in a malaria-endemic country, performed similarly to our Ugandan standard, although they only excluded clinical malaria and asymptomatic parasitemia by blood smear and rapid diagnostic testing (RDT) [20]. However, there was a trend towards under diagnosis of SGA which may be a limitation of the small sample size. We found that 74% of cases with asymptomatic parasitemia also had placental malaria. Based on these findings, assessing for asymptomatic parasitemia may be an effective proxy for placental malaria in relation to creating healthy population standards for neonatal growth. This result is consistent with prior studies that demonstrated an association between peripheral parasitemia during pregnancy and placental malaria [12,31]. Positive testing with RDT, used to assess parasitemia in the Schmiegelow study, has been found to be strongly associated with placental malaria [12,31]. Based on these results, it may be reasonable to utilize RDT to assess for parasitemia as a proxy for placental malaria, but future studies with larger sample sizes are needed.

### Future research directions

Our findings highlight the need for further research into a global birth weight reference or standard that is based on a diverse global population and is validated in cohorts from various high-, middle-, and low-income countries. Additionally, a larger cohort of placenta malaria negative neonates is needed to assess for differences that our small sample size may be underpowered to evaluate. Also, robust data from prospective fetal ultrasound data in well-characterized populations is needed to generate ideal fetal growth curves, which can potentially identify at risk pregnancies. Lastly, learning more about what factors contribute to normal fetal growth trajectory, with data on fetal ultrasound measurements during pregnancy, is needed to better assess deviations from the norm and subsequently identify potential identification markers.

### Strengths and limitations

Our study was limited by a small population size and we may have been underpowered to find differences between similarly performing birth weight standards and references. All of the patients in our database were taking intermittent preventative therapy in pregnancy (IPTp) for malaria and thus, we may have underestimated the true prevalence of placental malaria in the general population where IPTp uptake is not 100%. Additionally, because women in this study volunteered to be involved in these trials, there may an element of selection bias that may limit the generalizability of our outcomes. Because the study was based upon birthweights, it is unclear how well these values will translate to estimated fetal weights and characterize fetal growth restriction. Future research is needed in this area.

To our knowledge, this is the first study to strategically create a Sub-Saharan birth weight standard based on obstetrical ultrasound dating that excluded maternal malarial infection over the course of pregnancy in HIV-negative women. The women were closely followed from 12–20 weeks through delivery. Because of the prospective collection of data, we were able to create a Ugandan standard based on a true healthy reference population.

### Conclusions

Our findings, along with the current literature, highlight the importance of utilizing healthy populations to establish reference standards. Excluding women with malaria in malaria-endemic areas, creates birth weight norms at higher values and increases the detection of SGA. We found that excluding malaria does not have to be as extensive as doing placental pathology, but should include assessment of clinical malaria and asymptomatic parasitemia. Ensuring a true healthy population reference will better identify neonates who are pathologically small and require intervention.

## Supporting information

S1 Table Sample size per gestational age (completed weeks). (DOCX)

S1 Dataset (XLSX)

## References

[1] Lee ACC, Kozuki N, Cousens S, Stevens GA, Blencowe H, Silveira MF, et al Estimates of burden and consequences of infants born small for gestational age in low and middle income countries with INTERGROWTH-21 st standard: Analysis of CHERG datasets. BMJ. 2017 10.1136/bmj.j3677 28819030 PMC5558898

[2] Katz J, Wu LA, Mullany LC, Coles CL, Lee ACC, Kozuki N, et al Prevalence of small-for-gestational-age and its mortality risk varies by choice of birth-weight-for-gestation reference population. PLoS One. 2014 10.1371/journal.pone.0092074 24642757 PMC3958448

[3] Kiserud T, Piaggio G, Carroli G, Widmer M, Carvalho J, Neerup Jensen L, et al The World Health Organization Fetal Growth Charts: A Multinational Longitudinal Study of Ultrasound Biometric Measurements and Estimated Fetal Weight. PLoS Med. 2017 10.1371/journal.pmed.1002220 28118360 PMC5261648

[4] Natale V, Rajagopalan A. Worldwide variation in human growth and the World Health Organization growth standards: A systematic review. BMJ Open. 2014 10.1136/bmjopen-2013-003735 24401723 PMC3902406

[5] Buck Louis GM, Grewal J, Albert PS, Sciscione A, Wing DA, Grobman WA, et al Racial/ethnic standards for fetal growth: The NICHD Fetal Growth Studies. American Journal of Obstetrics and Gynecology. 2015 10.1016/j.ajog.2015.08.032 26410205 PMC4584427

[6] Papageorghiou AT, Ohuma EO, Altman DG, Todros T, Ismail LC, Lambert A, et al International standards for fetal growth based on serial ultrasound measurements: The Fetal Growth Longitudinal Study of the INTERGROWTH-21st Project. Lancet. 2014 10.1016/S0140-6736(14)61490-2 25209488

[7] Campbell MC, Tishkoff SA. African Genetic Diversity: Implications for Human Demographic History, Modern Human Origins, and Complex Disease Mapping. Annu Rev Genomics Hum Genet. 2008 10.1146/annurev.genom.9.081307.164258 18593304 PMC2953791

[8] Verhoeff FH, Brabin BJ, Van Buuren S, Chimsuku L, Kazembe P, Wit JM, et al An analysis of intra-uterine growth retardation in rural Malawi. Eur J Clin Nutr. 2001 10.1038/sj.ejcn.1601200 11477467

[9] Williams RL, Creasy RK, Cunningham GC, Hawes WE, Norris FD, Tashiro M. Fetal growth and perinatal viability in california. Obstet Gynecol. 1982 7070736

[10] American College of Obstetricians and Gynecologists, American Institute of Ultrasound in Medicine, Society for Maternal-Fetal Medicine. Committee Opinion No. 611. Method for Estimating Due Date. Obstet Gynecol. 2014 10.1097/01.AOG.0000454932.15177.be 25244460

[11] Zakama AK, Gaw SL. Malaria in Pregnancy: What the Obstetric Provider in Nonendemic Areas Needs to Know. Obstet Gynecol Surv. 2019;74: 546–556. 10.1097/OGX.0000000000000704 31830300 PMC7560991

[12] Kapisi J, Kakuru A, Jagannathan P, Muhindo MK, Natureeba P, Awori P, et al Relationships between infection with Plasmodium falciparum during pregnancy, measures of placental malaria, and adverse birth outcomes NCT02163447 NCT. Malar J. 2017;16: 1–11. 10.1186/s12936-016-1650-6 28982374 PMC5629777

[13] Rogerson SJ, Hviid L, Duffy PE, Leke RF, Taylor DW. Malaria in pregnancy: pathogenesis and immunity. Lancet Infect Dis. 2007;7: 105–117. 10.1016/S1473-3099(07)70022-1 17251081

[14] Lufele E, Umbers A, Ordi J, Ome-Kaius M, Wangnapi R, Unger H, et al Risk factors and pregnancy outcomes associated with placental malaria in a prospective cohort of Papua New Guinean women. Malar J. 2017 10.1186/s12936-017-2077-4 29065884 PMC5655867

[15] Fitri LE, Sardjono TW, Rahmah Z, Siswanto B, Handono K, Dachlan YP. Low fetal weight is directly caused by sequestration of parasites and indirectly by IL-17 and IL-10 imbalance in the placenta of pregnant mice with malaria. Korean J Parasitol. 2015 10.3347/kjp.2015.53.2.189 25925177 PMC4416375

[16] Ismail MR, Ordi J, Menendez C, Ventura PJ, Aponte JJ, Kahigwa E, et al Placental pathology in malaria: A histological, immunohistochemical, and quantitative study. Hum Pathol. 2000 10.1016/S0046-8177(00)80203-8 10665918

[17] Moya-Alvarez V, Abellana R, Cot M. Pregnancy-associated malaria and malaria in infants: An old problem with present consequences. Malar J. 2014;13: 1–10. 10.1186/1475-2875-13-1 25015559 PMC4113781

[18] Duffy PE. Plasmodium in the placenta: Parasites, parity, protection, prevention and possibly preeclampsia. Parasitology. 2007 10.1017/S0031182007000170 17958923

[19] Mikolajczyk RT, Zhang J, Betran AP, Souza JP, Mori R, Gülmezoglu AM, et al A global reference for fetal-weight and birthweight percentiles. Lancet. 2011 10.1016/S0140-6736(11)60364-4 21621717

[20] Schmiegelow C, Scheike T, Oesterholt M, Minja D, Pehrson C, Magistrado P, et al Development of a Fetal Weight Chart Using Serial Trans-Abdominal Ultrasound in an East African Population: A Longitudinal Observational Study. PLoS One. 2012 10.1371/journal.pone.0044773 23028617 PMC3448622

[21] Kakuru A, Jagannathan P, Muhindo MK, Natureeba P, Awori P, Nakalembe M, et al Dihydroartemisinin–Piperaquine for the Prevention of Malaria in Pregnancy. N Engl J Med. 2016;374: 928–939. 10.1056/NEJMoa1509150 26962728 PMC4847718

[22] Kajubi R, Ochieng T, Kakuru A, Jagannathan P, Nakalembe M, Ruel T, et al Monthly sulfadoxine–pyrimethamine versus dihydroartemisinin–piperaquine for intermittent preventive treatment of malaria in pregnancy: a double-blind, randomised, controlled, superiority trial. Lancet. 2019 10.1016/S0140-6736(18)32224-4 30910321

[23] Briggs J, Ategeka J, Kajubi R, Ochieng T, Kakuru A, Ssemanda C, et al Impact of Microscopic and Submicroscopic Parasitemia during Pregnancy on Placental Malaria in a High-Transmission Setting in Uganda. J Infect Dis. 2019;220: 457–466. 10.1093/infdis/jiz130 30891605 PMC6941619

[24] Rogerson SJ, Pollina E, Getachew A, Tadesse E, Lema VM, Molyneux ME. Placental monocyte infiltrates in response to Plasmodium falciparum malaria infection and their association with adverse pregnancy outcomes. Am J Trop Med Hyg. 2003 10.4269/ajtmh.2003.68.1.0680115 12556159

[25] Platt RW. The effect of gestational age errors and their correction in interpreting population trends in fetal growth and gestational age-specific mortality. Semin Perinatol. 2002 10.1053/sper.2002.34768 12211621

[26] Landis SH, Ananth C V., Lokomba V, Hartmann KE, Thorp JM, Horton, et al Ultrasound-derived fetal size nomogram for a sub-Saharan African population: A longitudinal study. Ultrasound Obstet Gynecol. 2009 10.1002/uog.6357 19402076

[27] Gardosi J, Francis A. A customized standard to assess fetal growth in a US population. Am J Obstet Gynecol. 2009 10.1016/j.ajog.2009.04.035 19576371

[28] Gardosi J, Francis A, Turner S, Williams M. Customized growth charts: rationale, validation and clinical benefits. American Journal of Obstetrics and Gynecology. 2018 10.1016/j.ajog.2017.12.011 29422203

[29] Chiossi G, Pedroza C, Costantine MM, Truong VTT, Gargano G, Saade GR. Customized vs population-based growth charts to identify neonates at risk of adverse outcome: systematic review and Bayesian meta-analysis of observational studies. Ultrasound in Obstetrics and Gynecology. 2017 10.1002/uog.17381 27935148

[30] Francis A, Hugh O, Gardosi J. Customized vs INTERGROWTH-21 st standards for the assessment of birthweight and stillbirth risk at term. Am J Obstet Gynecol. 2018;218 10.1016/j.ajog.2017.12.013 29422208

[31] De Beaudrap P, Turyakira E, White LJ, Nabasumba C, Tumwebaze B, Muehlenbachs A, et al Impact of malaria during pregnancy on pregnancy outcomes in a Ugandan prospective cohort with intensive malaria screening and prompt treatment. Malar J. 2013 10.1186/1475-2875-12-139 23617626 PMC3642015

