# Supplementary material for: Generation of a malaria negative Ugandan birth weight standard for the diagnosis of small for gestational age
Source: PLoS One. 2020 Oct 2;15(10):e0240157. doi: 10.1371/journal.pone.0240157 (PMC7531849; doi:10.1371/journal.pone.0240157)
Supplement: S1 Table — (DOCX) [file pone.0240157.s001.docx]

S1 Table. Sample size per gestational age (completed weeks)

| Any malaria | | Malaria Negative | |
| --- | --- | --- | --- |
| GA | Sample size (n) | GA | Sample size (n) |
| 28 | 1 | 28 | 0 |
| 29 | 2 | 29 | 0 |
| 30 | 0 | 30 | 1 |
| 31 | 4 | 31 | 0 |
| 32 | 0 | 32 | 2 |
| 33 | 5 | 33 | 5 |
| 34 | 4 | 34 | 2 |
| 35 | 10 | 35 | 2 |
| 36 | 13 | 36 | 9 |
| 37 | 22 | 37 | 27 |
| 38 | 86 | 38 | 70 |
| 39 | 150 | 39 | 94 |
| 40 | 140 | 40 | 119 |
| 41 | 81 | 41 | 51 |
| 42 | 10 | 42 | 10 |
| 43 | 5 | 43 | 0 |
| 44 | 0 | 44 | 0 |
| 45 | 0 | 45 | 1 |
| GA: gestational age (based on completed weeks) | | | |
